# Herpes zoster risk after 21 specific cancers: population-based case–control study

**DOI:** 10.1038/bjc.2017.124

**Published:** 2017-05-02

**Authors:** Erik Hansson, Harriet J Forbes, Sinéad M Langan, Liam Smeeth, Krishnan Bhaskaran

**Affiliations:** 1 Department of Non-Communicable Diseases Epidemiology, London School of Hygiene and Tropical Medicine, Keppel Street, London, WC1E 7HT, UK

**Keywords:** herpes zoster, malignancy, cancer, CPRD, haematology, oncology, shingles

## Abstract

**Background:**

Some malignancies are known to be associated with increased risk of herpes zoster, but little is known about how associations between cancer and subsequent zoster risk vary by cancer site, by time since cancer diagnosis, and by age.

**Methods:**

An age-, sex-, calendar time-, and practice-matched case–control study, nested in the broadly UK representative Clinical Practice Research Datalink (CPRD) primary care database, was analysed using conditional logistic regression to estimate the association between 21 of the most common specific malignancies and subsequent zoster risk. We adjusted for comorbid conditions and other potential confounders, and investigated effect modification by age and time since malignancy diagnosis.

**Results:**

A total of 192 081 adult zoster patients and 732 035 controls were included. Malignancy overall was positively associated with zoster risk (adjusted OR 1.29, 95% CI 1.27–1.32), and the association was especially strong for haematological malignancies (OR 2.46, 2.33–2.60). Among specific malignancies, there was evidence that oral, oesophageal, stomach, colorectal, lung, breast, ovarian, prostate, kidney, bladder, and CNS cancers, as well as lymphoma, myeloma, and leukaemia were associated with increased zoster odds (*P*⩽0.05 in each case), but the magnitude of associations varied widely. The association was typically strongest within 2 years of malignancy diagnosis and decreased with older age for both haematological and solid malignancies.

**Conclusions:**

Several cancers were associated with an increased risk of zoster, particularly within the first 2 years after diagnosis and among younger individuals. Knowledge that patients with a recent diagnosis of cancer are at high risk of zoster may encourage initiation of antiviral therapy earlier in the course of zoster when the benefits are greater. Evaluation of whether patients diagnosed with cancer would benefit from early zoster vaccination is warranted.

Herpes zoster (shingles) occurs when varicella zoster virus (VZV) reactivates from its dormant state during periods of reduced cell-mediated immunity, causing a characteristic rash and substantial pain (Thomas and Hall, 2004), which can significantly impact on quality of life (Drolet *et al*, 2010). Zoster incidence is 3–5 per 1000 person years and 5–30% of patients develop postherpetic neuralgia (PHN; Kawai *et al*, 2014). A vaccine to prevent zoster has recently become available, but its high cost makes identifying priority groups important. Zoster incidence is increased among females and older individuals (Thomas and Hall, 2004; Kawai *et al*, 2014; Liu *et al*, 2015), and with many immunosuppressed conditions (Forbes *et al*, 2014).

Cancer patients may experience cell-mediated immunosuppression, resulting from chemotherapy, psychological stress, or physical trauma of surgery or radiotherapy, putting them at greater risk of herpes zoster. We identified seven studies estimating an association between prevalent/previous malignancy and zoster (Heymann *et al*, 2008; Hata *et al*, 2011; Habel *et al*, 2013; Weitzman *et al*, 2013; Forbes *et al*, 2014; Liu *et al*, 2015; Yenikomshian *et al*, 2015), and all of them found a positive association. Studies of specific malignancies and zoster are important for informing prevention and understanding causality but have been underpowered (Habel *et al*, 2013), had control groups severely impacting on interpretability (Hata *et al*, 2011; Habel *et al*, 2013), or analysed a limited number of specific malignancies (Heymann *et al*, 2008; Forbes *et al*, 2014) or sub-groups (Yenikomshian *et al*, 2015).

Only a few studies have looked at interactions with age and time since malignancy diagnosis (Heymann *et al*, 2008; Habel *et al*, 2013; Weitzman *et al*, 2013; Yenikomshian *et al*, 2015); further evidence on this is needed to identify when efforts to prevent malignancy-related zoster are likely to be most beneficial.

We undertook the present study to overcome the above-listed limitations of existing evidence by performing well-powered analyses investigating the associations between a range of site-specific malignancies, some of which have not been studied previously, and herpes zoster, in the general population, and exploring how these associations vary by age and time since cancer diagnosis.

## Materials and Methods

### Study design

An age-, sex-, calendar time-, and practice-matched case–control study, nested within the Clinical Practice Research Datalink (CPRD) cohort was used. The study is based on a previous published study of zoster risk factors (Forbes *et al*, 2014), which provides further details on participant selection and variable definitions, to supplement our description below.

### Setting

Clinical Practice Research Datalink is a database of general practice patient records covering 7% of the 98% of the UK population registered with a general practitioner (GP). It is broadly representative of this population in terms of age, sex, and ethnicity (Herrett *et al*, 2015). It was initially set up in 1987 and contains GP diagnoses and prescriptions, and information sent from hospitals to GPs in response to referrals in Read code format. 58% of CPRD participants are linked to the Hospital Episode Statistics (HES) database, storing International Classification of Disease (ICD) codes from hospital attendance (Herrett *et al*, 2015). Linkage is only available for participants in England, but there are reported to be no substantial differences in patient characteristics between those that are linked to HES and those that are not (Gallagher *et al*, 2011). Varicella vaccination is not given in the UK, but zoster vaccination was initiated for those aged 70 and 79 years in September 2013.

### Participants

The base study population from which the cases and controls were selected consisted of individuals in CPRD with and without HES linkage, in follow-up any time between 1 January 2000 and 30 August 2015, >18 years, with no historical records for diagnosed zoster or PHN before start of follow-up in the database. To exclude the possibility that zoster vaccination influenced the results, a sensitivity analysis was conducted restricted to zoster cases diagnosed up to September 2013, which is the date that vaccination was introduced in the UK.

Cases were those with an incident herpes zoster diagnosis (ICD10 code B02, B02.0, B02.1, B02.31, B02.7, B02.8, B02.9, or G53.0 in HES or corresponding Read codes in CPRD (Supplementary Appendix A)) recorded in CPRD or HES after at least 12 months of follow-up in CPRD. This 12-month criterion was used to ensure that cases were truly incident, since zoster coded early in follow-up might represent retrospective recording of clinical history in the period immediately after GP registration (Lewis *et al*, 2005). Patients identified through HES with zoster diagnosis codes in the secondary diagnosis field were excluded, as it was uncertain if this reflected incident zoster. The earliest date of either the CPRD or HES zoster diagnosis date was used as the index date.

Controls were sampled using incidence density sampling. They were required to have no previous zoster diagnosis recorded at the index date of their matched case; as with cases, controls were also required to have at least twelve months of follow-up preceding the index date, which would allow time for any historical zoster episodes to be recorded by the GP.

### Variables

Information on comorbidities, alcohol use, smoking, body mass index (BMI), and GP prescription records of oral and inhaled corticosteroids, and other immunosuppressive treatment within three months preceding index date was obtained through CPRD. Three months was considered a reasonable cutoff considering the assumed duration of immunosuppressive effects of these drugs (Forbes *et al*, 2014). All explanatory variables were obtained using the closest relevant records prior to the index date for both cases and controls, except for BMI, which could be obtained within 1 year after index date, if no prior records were available.

First malignancy diagnosis was obtained by searching clinical records for Read codes mapping to cancer diagnoses; our methodology for identifying cancer Read codes and mapping them to ICD-10 malignancy codes has been previously described (Bhaskaran *et al*, 2014). The 21 malignancies that were most prevalent in the data set were studied (oral (ICD10 code C00–06), salivary (C07–08), oesophageal (C15), stomach (C16), colorectal (C18–20), larynx (C32), lung (C34), melanoma (C43), breast (C50), cervical (C53), uterus (C54–55), ovarian (C56), prostate (C61), testicular (C62), kidney (C64), bladder (C67), CNS (C71–72), and thyroid (C73) cancer, and lymphoma (C81–84), myeloma (C90) and leukaemia (C91–95)). Only the first malignancy diagnosis was obtained, as determining whether further malignancy diagnoses were metastases of the first or new malignancies was infeasible. Participants could therefore only be considered exposed to one malignancy. A total of 17% of all solid malignancy diagnoses could not be attributed to any of the 18 most prevalent site-specific solid malignancies—12% because the Read code did not specify the site of the cancer, and 5% because they referred to rarer cancers. In total, 5% of all haematological malignancies could not be sub-classified further. Non-melanoma skin malignancies were not considered. As a *post hoc* analysis, leukaemia was subdivided into acute, chronic and unspecified/other. Cancer diagnoses could be dated earlier than the start of CPRD since we assumed that a previous cancer record would have been transferred into the CPRD record when the patient registered with a GP connected to CPRD. However, in case of incomplete capture of cancer prior to CPRD follow-up, we did a sensitivity analysis, restricting the study population to those who had no recorded history of cancer up to 12 months after the start of CPRD follow-up, and then restricting the time window for defining cancer exposure to the period after this date. As another sensitivity analysis, regular use of aciclovir (defined as having at least two aciclovir prescriptions from a GP <6 weeks apart, any time between CPRD follow-up start and index date) was adjusted for, to address potential confounding by such medication.

### Statistical methods

The explanatory variables were described by case–control status.

#### Association between previous malignancy and zoster risk

Conditional logistic regression was used to estimate the OR with 95% confidence intervals (CIs) for incident zoster after malignancy diagnosis. Due to the matching, all analyses accounted for the matching variables age, sex, practice, and calendar time. The exposure (prevalent/previous malignancy) was considered at increasing levels of detail; (1) any malignancy diagnosis, (2) solid or haematological malignancy, and (3) site-specific solid malignancy or specific haematological malignancy diagnosis. All ORs represent comparisons to the reference group of those without a malignancy diagnosis.

The three levels of exposure detail described above were analysed adjusting for covariates in three stages; (i) adjusting only for matching factors, (ii) adjusting also for rheumatoid arthritis (RA), systemic lupus erythematosus (SLE), inflammatory bowel disease (IBD), COPD, asthma, chronic kidney disease (CKD), depression, diabetes (by type), HIV, other cellular immunodeficiency (OID), alcohol use, smoking, BMI, and inhaled corticosteroid use as these were considered *a priori* potential confounders, and (iii) adjusting additionally for haematopoietic stem cell transplant (HSCT), oral corticosteroid use and other immunosuppressive therapy prescribed by GPs to explore if the association was mediated by these. Participants with missing data on any explanatory variables were excluded from all analyses, known as a complete case analysis, which is valid if missingness is independent of the outcome (zoster), conditional on all covariates (White and Carlin, 2010). Likelihood ratio tests were used to assess the evidence of an association between exposure and outcome.

#### Variation by time since cancer diagnosis

For the malignancies that were associated with zoster in the main analysis, we estimated the OR of zoster according to time since malignancy diagnosis (<1, 1–2, 2–3 and >3 years before index date, compared to no prior malignancy). Only matched factors were adjusted for to avoid precision loss arising from excluding cases with missing data, and because our earlier analysis suggested limited confounding; however, in a sensitivity analysis we adjusted for all potential confounders as in the main model. The strength of the evidence of temporal variation in odds of zoster after malignancy diagnosis was assessed using the likelihood ratio test. A test for linear trend in the zoster OR with time since malignancy diagnosis was performed by comparing a model with a binary malignancy variable to a model that included years since diagnosis. The likelihood value obtained in a model including only the malignancy as a binary ‘ever *vs* never’ malignancy diagnosis was compared to one including instead a four-level variable describing the time since diagnosis of that malignancy (1, 2, 3, >3 years) entered as a linear term. This was done for each of the malignancy types, one at a time. To test for a non-linear trend, a model with time since malignancy diagnosis entered as a linear term was compared to one with time since malignancy diagnosis entered as a categorical term. Again, these tests were repeated for each specific malignancy.

#### Variation by age

Interaction between age at index date and malignancies was assessed by including interaction parameters between age at index date (<50, 50–60, 60–70, 70–80, and >80 years) and malignancies (solid/haematological), adjusting for all potential confounders and using likelihood ratio tests to assess the strength of the evidence of age interaction. This test was performed by comparing the likelihood of a model including an age-malignancy interaction variable with one including age and malignancy entered as independent covariates.

### Ethical approval

This study used non-identifiable data routinely gathered through existing infrastructure (CPRD and HES) within the health-care system and informed consent for this study was not needed. The study received ethical approval from the LSHTM Ethics Committee on the 22 April 2016 (Reference number 11200); the study was also approved by the CPRD Independent Scientific Advisory Committee (approval number 16_113A; protocol supplied as Supplementary Appendix B).

## Results

**Figure 1:**
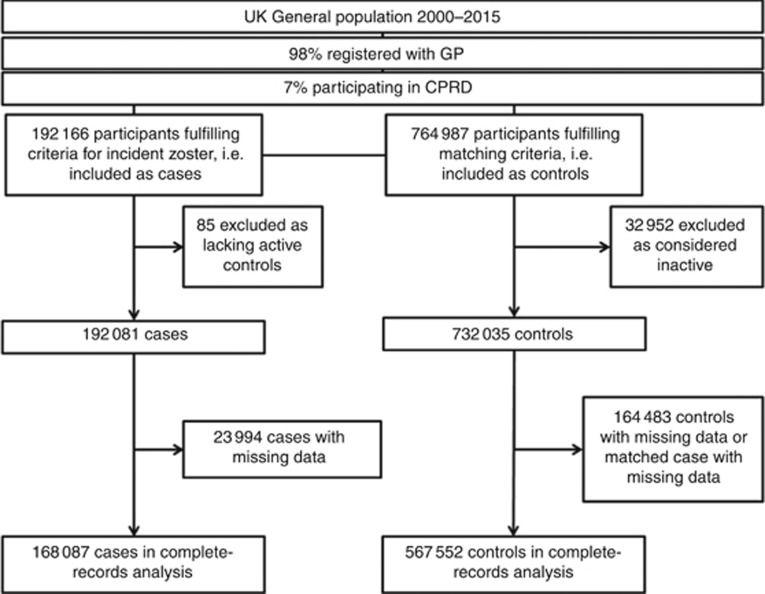
**Participant flow chart.**

A total of 192 081 participants fulfilled the criteria for being considered incident cases of zoster and had active matched controls, and 732 035 were identified as controls for these cases (Figure 1). Table 1 describes the potential confounders and effect mediators by case–control status. The median age of cases and controls at index date was similar as expected due to the matching (62.3 years, interquartile range (IQR) 49.3–73.8 years for cases; 61.8 years, IQR 48.5–73.6 years for controls). The median time from malignancy diagnosis until zoster was 6.1 years (IQR 2.4 to 12.3 years). The majority of cases (61%) and controls (59%) were women. Asthma, type 2 diabetes, CKD, COPD, RA, SLE and IBD, OID, HSCT and HIV were more common among cases than controls, as was GP prescription of corticosteroids and other immunosuppressive medications. Supplementary Appendix C describes the prevalence of malignancy among controls by covariates.

There were missing data on BMI, smoking and alcohol for a total of 14% of the participants. Cases less frequently had any missing data (12%), as did those with malignancies (10% Supplementary Appendix D).

### Association between previous malignancy and zoster risk

A total of 192 081 patients had a diagnosis of incident zoster during follow-up, and 16 219 (8.4%) of these had any type of previous malignancy diagnosis at the date of zoster diagnosis, as compared to 48 704 (6.7%) of controls.

Table 2 shows the associations between previous/prevalent malignancy and incident herpes zoster in models with different levels of covariate adjustment. There was little change in the estimated associations between the models accounting for matching factors only (Table 2, ‘Model 1’), and the models adjusting for *a priori* potential confounders (Table 2, ‘Model 2’). In the adjusted models, patients with any previous/prevalent malignancy had 1.29 (95% confidence interval (CI) 1.27–1.32) times higher zoster odds overall than those without such diagnosis. The OR for the association between haematological malignancies and zoster was 2.46 (95% CI 2.33–2.60), and 1.19 (95% CI 1.17–1.22) for solid malignancies.

**Figure 2:**
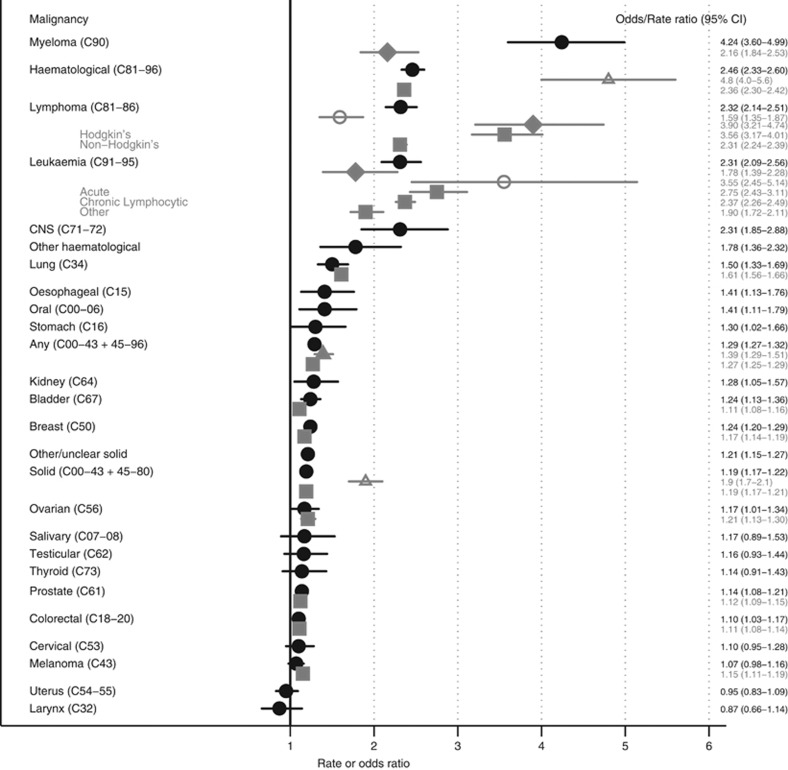
**Odds ratios of zoster for different malignancy diagnoses adjusted for potential confounders, compared to previous studies.** Black filled circles—present study estimates. Previous studies in grey: Filled triangle—(Liu *et al*, 2015), square—(Yenikomshian *et al*, 2015), hollow triangle—(Habel *et al*, 2013) hollow circle—(Heymann *et al*, 2008), diamond—(Forbes *et al*, 2014).

For 11 of the 18 specific solid (non-haematological) cancers considered, we found evidence of a positive association with odds of subsequent zoster in adjusted models (Table 2 ‘Model 2’, and Figure 2). The largest association was between CNS cancer and zoster (adjusted OR=2.31, 95% CI 1.85–2.88); followed by lung, oral and oesophageal cancers (adjusted OR=1.50, (1.33–1.69); 1.41 (1.11–1.79) and 1.41 (1.13–1.76), respectively). More modest associations were seen for stomach, colorectal, breast, ovarian, prostate, kidney and bladder cancers (adjusted ORs in the range 1.10 to 1.30). There was little or no evidence that salivary, larynx, cervix, uterus, testicular, or thyroid cancers, or melanoma were associated with zoster; though in each case, confidence intervals did not exclude a small positive association. There was no evidence that any cancer was inversely associated with zoster. Among the haematological malignancies, there was strong evidence that lymphoma, myeloma, and leukaemia were all associated with a more than doubling in the odds of zoster; myeloma was associated with the greatest increase in odds (adjusted OR=4.24 (3.60–4.99)).

Adjusting additionally for covariates potentially on the causal pathway (HSCT, and oral corticosteroid and immunosuppression prescribed by GPs) resulted in only very slight decreases in most estimates of exposure OR, at most a 14% decrease for CNS cancer, from 2.31 to 2.12 (Table 2, ‘Model 3’).

### Variation by time since cancer diagnosis

**Figure 3:**
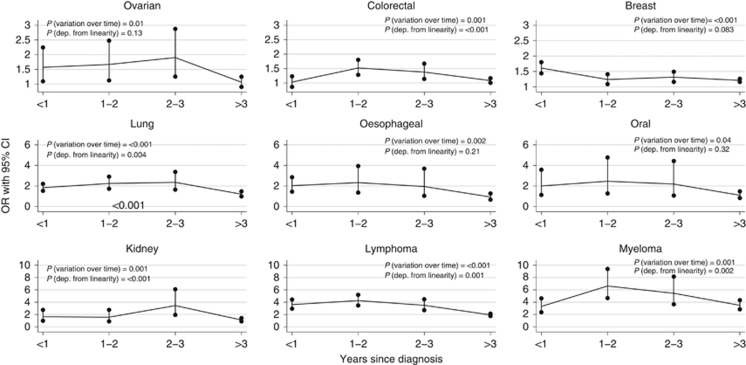
**Zoster odds ratio by time since malignancy diagnoses.** Numerical results are available in Supplementary Appendix E. Note: panels have different y-scales.

There was good evidence that the changes in risk of zoster associated with oral, oesophageal, colorectal, lung, breast, ovarian and kidney cancer, lymphoma, and myeloma changed according to time since the cancer diagnosis (*P*<0.05 in each case), but there was no evidence that the associations of stomach, prostate, bladder or CNS cancers or leukaemia with zoster changed over time (Figure 3; Supplementary Appendix E). For colorectal, lung, and kidney cancer, lymphoma and myeloma, the associations varied non-linearly after diagnosis, with peaking zoster OR 1–3 years after malignancy diagnosis; for oral, oesophageal, ovarian and breast cancer, there was no evidence that the association was non-linear (*P* for departure from linearity>0.05), with zoster odds generally decreasing with time since diagnosis. Adjusting for potential confounders did not change the estimates substantially (Supplementary Appendix E).

### Variation by age

**Figure 4:**
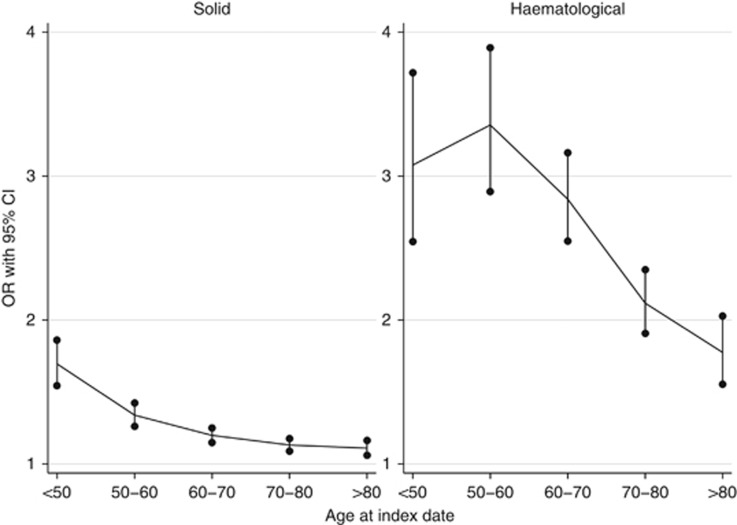
**Age-stratum specific odds ratios of herpes zoster among malignancy patients.** Numerical results are available in Supplementary Appendix F.

There was strong evidence of interaction between age at index date and malignancy (*P*<0.001; Figure 4, Supplementary Appendix F), with malignancies more strongly associated with zoster among younger participants (for solid malignancies OR=1.70 (1.54–1.86) among those <50 *vs* 1.11 (1.06–1.16) among those >80 years old, and 3.08 (2.55–3.72) *vs* 1.77 (1.55–2.03) for haematological).

Restricting the data set to before zoster vaccination commenced in September 2013 did not affect the results importantly, but restricting the data set to those without a malignancy diagnosis until after 12 months of follow-up in CPRD led to generally increasing strength of association (Supplementary Appendix G). Aciclovir was prescribed regularly by a GP among 0.5% of zoster cases and 0.2% of controls, but there were no important changes in estimated associations between cancer and zoster when such prescription was adjusted for (Supplementary Appendix G). Acute and chronic leukaemia were both similarly associated with zoster, and there was no evidence of difference in the timing of excess zoster risk after a diagnosis of either of them (*P*=0.97 and 0.89, respectively, Supplementary Appendix H).

## Discussion

### Key results

Having a previous cancer diagnosis was associated with a modest, but clear increase in the odds of zoster, but there was substantial heterogeneity in the association according to type of malignancy. The largest associations were observed between haematological malignancies and subsequent odds of zoster, with lymphoma and leukaemia associated with a more than doubling in the odds of zoster in adjusted models, and myeloma associated with a 4.24-fold increase in odds. 11 out of 18 of the solid (non-haematological) cancers investigated were also positively associated with subsequent zoster risk; the largest association was with CNS cancer (adjusted OR=2.31), while oral, oesophageal, stomach, colorectal, lung, breast, ovarian, prostate, kidney, and bladder cancers were also each associated with 10–50% increases in odds of zoster. This association between cancer and zoster was larger among younger patients, and for several malignancies the association varied by time since diagnosis, typically peaking within 3 years after malignancy diagnosis.

### Strengths and limitations

To our knowledge, this is the largest study to date to look in comparable detail at the associations between a wide range of cancer types and subsequent zoster risk. The large study size enabled us to estimate associations with high precision, even for less common cancer types, which is a major strength of this study. However, the use of routinely collected data that enabled this large study size inevitably led to some limitations.

CPRD generally has high validity (Herrett *et al*, 2010), but no known study has validated CPRD zoster diagnosis specifically. Our outcome was not based on standardised diagnostic criteria, which is a limitation, and there is no information available on how each zoster diagnosis was made, but in clinical practice in the UK, zoster is usually diagnosed based on observing a characteristic unilateral rash with dermatomal distribution rather than laboratory testing (Forbes *et al*, 2014), as per recent guidelines (Werner *et al*, 2017).

Outside CPRD, two studies have compared clinical zoster diagnosis to serological (Opstelten *et al*, 2007) and PCR (Tseng *et al*, 2013) verified diagnosis and found a positive predictive value (PPV) of 91% and 85%, respectively. Four studies (Donahue *et al*, 1995; Heymann *et al*, 2008; Yawn *et al*, 2011; Habel *et al*, 2013) estimated the PPV of electronic database zoster diagnosis by comparing findings to review of patient records, reporting PPVs between 69 and 87%. Two studies using either questionnaire or PHN diagnosis estimated that 95 (Lu *et al*, 2009) and 81% (Weitzman *et al*, 2013) of patients respectively sought health care for zoster. The extent of incorrect rejection of herpes zoster diagnosis in primary care has, to our knowledge, not been studied, but there are only a few rare clinical conditions with which the characteristic clinical appearances could be confused. The most common potential misdiagnosis is herpes simplex, but this only rarely presents with a similar, dermatomal distribution (Rubben *et al*, 1997). Considering that zoster is usually easy to diagnose clinically, the proportion of zoster cases or controls that are misclassified is likely to be small. There is no clear reason why malignancy would cause a clinician to be more or less likely to diagnose a zoster-like rash as zoster, so any misclassification is likely to be non-differential, thereby biasing the results towards null (Forbes *et al*, 2014). Studies using cases confirmed through patient record review (Hata *et al*, 2011; Habel *et al*, 2013), found stronger associations with malignancies, but these estimates may be biased upwards if the record reviewers were not (reportedly) blind to exposure status.

Validity of CPRD cancer recording has been investigated via concordance with national registration and hospital data; >90% of CPRD cancers were confirmed in other data sources, and >90% of nationally registered cancers were present in CPRD, with CPRD missing mainly rapidly fatal malignancies (Boggon *et al*, 2013). Missing such cancers would be expected to lead to a bias towards the null, but such cases are likely to be rare, so this should not have had a major impact on our results. Twelve per cent of cancers were of unspecified site, these may have included metastatic cancers with unknown primaries, or cancers with imperfect coding of cancer site (e.g., site details may have been recorded in non-coded free text). As patients with these ‘other/unclear solid’ malignancies may have had more advanced cancer at diagnosis, they may have been generally sicker and at higher zoster risk, causing bias towards null in the associations with specific malignancies that are consequently misclassified. However, the OR for ‘other/unclear solid malignancy’ was virtually identical to that for ‘any solid malignancy’ suggesting that any such bias is likely to have been of small magnitude.

The magnitude of association increased in a sensitivity analysis that excluded those with a prevalent malignancy diagnosis at start of CPRD follow-up (defined as any diagnosis recorded before 12 months of complete follow-up in CPRD). This change would be consistent with incomplete capture of pre-follow-up (i.e., historical) malignancy diagnosis in the primary analysis, leading to a bias towards the null. However, the estimated associations were qualitatively similar and the changes in estimates were modest compared to the main analysis, suggesting that this information bias had only limited impact.

More health-care contact among patients with a prior cancer diagnosis could have led to a greater opportunity to diagnose zoster early. Patients with malignancies had a similar mean consultation rate as those with epilepsy (18.4 *vs* 19.6 consultations per year), for whom only a very small increase in odds of zoster has been found (OR=1.05, 99% CI 0.97–1.14; Forbes *et al*, 2014), indicating that if there is ascertainment bias due greater GP consultation, this is likely to have had only limited impact. Controls were required to have recent GP contact, which may have disproportionally excluded healthy controls and introduced bias towards null (Forbes *et al*, 2014). However, only 5% of controls were excluded due to inactivity, so the magnitude is likely small.

There were some missing data in lifestyle-related variables (BMI, smoking, alcohol); these were considered unlikely to be missing at random in a primary care setting (Bhaskaran and Smeeth, 2014), so we used a complete case analysis. Having missing data was inversely related to the outcome in unadjusted analysis but the assumption underlying complete case analysis, which relates to *conditional* independence between missingness and outcome, cannot be tested in the observed data due to the need to condition on the missing values themselves. Matched sets with missing data among cases are excluded, so that the consequences for analysis of missing data among cases is larger than among controls, likely creating bias away from zero as those with missing data were generally healthier. As only 14% of individuals overall had missing data this is unlikely to completely explain the observed associations.

Some potential confounders, such as ethnicity/race/country of origin and reproductive history were unavailable, but the *a priori* importance of these was considered quite limited. The covariates considered on the causal pathway (GP prescribed corticosteroids and other immunosuppressive drugs, adjusted for in ‘Model 3’) may have inadequately captured use of these drugs by cancer patients, who would more likely receive such treatments as part of hospital prescribing, which we could not measure; this may explain the minimal change in estimates when adjusting for these variables, limiting our ability to conclude definitively that receipt of these treatments did not mediate some of the associations seen. In addition to lacking information on surgery and chemo- and radiotherapy, information on antiviral prophylaxis is likely to be incomplete, as these treatments are delivered in secondary/tertiary care. Our sensitivity analysis on aciclovir use might therefore not truly capture confounding by use of this medication. Information on cancer stage could have been used as a proxy for what type of treatment a patient was receiving and thereby the extent of immunosuppression, but such information was unfortunately unavailable.

### Interpretation

These results strengthen the evidence that malignancies are associated with a higher zoster risk, congruent with previous studies (Figure 2; Heymann *et al*, 2008; Hata *et al*, 2011; Habel *et al*, 2013; Weitzman *et al*, 2013; Forbes *et al*, 2014; Liu *et al*, 2015; Yenikomshian *et al*, 2015). The discrepancies with estimates for haematological malignancies obtained using largely the same cohort previously (Forbes *et al*, 2014; Supplementary Appendix I), is likely attributable to the previous study using diagnoses within two years and less specific myeloma Read codes, for example, monoclonal gammopathy was coded as myeloma in this study, whereas the present study used only Read codes considered to be more definitely indicating myeloma.

Two broad potential mechanisms may explain an association between prevalent/previous malignancy and zoster. First, they may share the underlying cause of immunodysfunction, as both malignancies and zoster are favoured by a weakened immune system (Cotton *et al*, 2013); second, the malignant disease and/or its treatment may cause immune system dysfunction, causing zoster (Cotton *et al*, 2013; Habel *et al*, 2013). This study cannot differentiate between these mechanisms as information on immune system function and malignancy treatment is lacking. Haematological malignancies lead to severe immunosuppression from disease and its treatment, and are those with the strongest link to zoster, while malignancies such as melanoma, which is not treated with immunosuppressive therapy, are not as strongly associated with zoster, supporting the role of systemic chemotherapy. That CNS cancer patients have such comparatively high odds of zoster has, to our knowledge, not been described with such precision and adequate methodology previously. Plausibly, immune dysfunction specifically within the nervous system might increase the risk of both zoster and CNS cancer, or the treatment of CNS cancer might lead to a high risk of zoster, for example, through high-dose corticosteroids for decreasing oedema around intracranial tumours, chemotherapy able to cross the blood-brain barrier, or radiotherapy triggering zoster reactivation from cranial nerve nuclei.

Some studies considering history of zoster as a risk factor for malignancies (i.e., the opposite temporality) have found positive associations (Cotton *et al*, 2013) suggesting that the common-cause hypothesis is one valid explanation. The temporal variation of zoster odds after diagnosis indicates that something about the malignancy, rather than only a common cause (immunodeficiency) leads to an increased zoster risk, as immunodeficiency might be expected to be constant or deteriorate with time/age rather than recover spontaneously. For most malignancies, zoster odds ratio decreased with time, perhaps as an increasing proportion of surviving patients become cancer-free and are no longer being treated with potent immunosuppression. This finding also indicates that zoster vaccination of cancer patients might be most beneficial quite soon after diagnosis, if safety and effectiveness can be confirmed in this setting. The current, live zoster vaccine is contraindicated among the profoundly immunosuppressed, but efforts are underway to develop a subunit vaccine that could be useful among these patients. One previous study found a constantly increased zoster rate after malignancy diagnosis (Yenikomshian *et al*, 2015), whereas another found a pattern similar to our study (Weitzman *et al*, 2013).

The decreasing malignancy-zoster association with increased age may be due to absence of other zoster risk factors and/or more aggressive chemotherapy (Yenikomshian *et al*, 2015) among the young. These findings are consistent with previous studies (Heymann *et al*, 2008; Habel *et al*, 2013).

### Generalisability

As the study population is broadly representative of a high-income country, and the findings are congruent with studies from similar settings, the findings are likely to be generalisable to high-income countries. However, changes in cancer treatments, and the increasing popularity of immune-modulating cancer therapies mean that the associations we have observed between some cancers and zoster risk may change in the future.

### Recommendations

A recent diagnosis of cancer raises the index of clinical suspicion of zoster among patients presenting with characteristic initial symptoms. Such patients may benefit from rapid initiation of antiviral therapy, to reduce acute pain and complications, although strong evidence is lacking for a protective effect against PHN. Future studies should evaluate the safety, timing and cost-benefit balance for vaccinating cancer patients against zoster, considering each specific malignancy separately. To obtain maximum benefit, vaccination as soon as possible after cancer diagnosis may be needed, prior to immunosuppressive therapies being initiated. New vaccines, which are currently under development, may be available for immunosuppressed patients in the future. A previous study using CPRD found that, among patients with herpes zoster, those with a history of cancer have a similar risk of developing postherpetic neuralgia as other patients with no history of cancer (Forbes *et al*, 2016).

Future research should analyse zoster risk in relation to cancer therapy, malignancy stage and other detailed clinical information, to better understand the mechanisms explaining the association between cancer and zoster and how the excess risk may be prevented.

## Conclusions

Patients with malignancies are at increased zoster risk, with quite marked variation by type of cancer, possibly attributable to different treatment patterns. Risks were higher in the first few years post cancer diagnosis, and among younger people.

## Figures and Tables

**Table 1:** Distribution of covariates by case–control status

	**Controls**		**Cases**	
	***N***	**%**	***N***	**%**
Total number	732 035		192 081	
**Matching factors**				
Gender				
Male	286 486	39.1	78 011	40.6
Female	445 549	60.9	114 070	59.4
Age group				
18–29	52 190	7.1	14 517	7.6
30–49	138 077	18.9	37 554	19.6
50–59	137 975	18.8	36 310	18.9
60–69	162 446	22.2	41 540	21.6
70–79	147 765	20.2	37 547	19.5
80–89	82 390	11.3	21 182	11.0
90+	11 192	1.5	3431	1.8
**Adjusted for in step 2 (comorbidities and lifestyle factors)**				
Systemic lupus erythematosus	1139	0.2	537	0.3
Inflammatory bowel disease	7319	1.0	2589	1.3
Reumatoid arthritis	9689	1.3	3845	2.0
Chronic obstructive pulmonary disease	28 958	4.0	9683	5.0
Depression	29 136	4.0	8980	4.7
Asthma	37 004	5.1	11 673	6.1
Chronic kidney disease	48 959	6.7	14 358	7.5
HIV	159	0.0	189	0.1
Other cellular immunodeficiency	298	0.0	140	0.1
Inhaled corticosteroid therapy	46 013	6.3	15 172	7.9
Diabetes				
Type 1	1374	0.2	457	0.2
Type 2	55 492	7.6	14 974	7.8
Unknown	3011	0.4	1032	0.5
BMI category				
Underweight	14 263	1.9	3739	1.9
Normal weight	250 052	34.2	66 339	34.5
Overweight	240 445	32.8	64 464	33.6
Obese	155 263	21.2	41 659	21.7
Missing	72 012	9.8	15 880	8.3
Smoking				
Never-smoker	277 413	37.9	72 419	37.7
Current smoker	173 609	23.7	43 288	22.5
Ex-smoker	267 778	36.6	74 701	38.9
Missing	13 235	1.8	1673	0.9
Alcohol				
Never-drinker	72 345	9.9	18 147	9.4
Current drinker	515 761	70.5	137 716	71.7
Ex-drinker	68 986	9.4	19 161	10.0
Missing	74 943	10.2	17 057	8.9
**Adjusted for in step 3 (potential cancer treatment)**				
Other immunosuppressive treatment^a^	5075	0.7	2845	1.5
Oral corticosteroid treatment^a^	17 189	2.3	7901	4.1
Haematopoietic stem cell transplant	29	0.0	54	0.0

Abbreviation: BMI=body mass index.

Note. Patients may have many comorbidities and treatments.

a Other classifications than those used in original publication (Forbes *et al*, 2014), and these labels are mixed up in this publication.

**Table 2:** Prevalence of malignancies among cases and controls, and association between prevalent/previous malignancy and incident zoster

	**Controls**	**Cases**	**Model 1**^a^		**Model 2**^b^		**Model 3**^c^	
**Malignancy type**	***N*** **(%)**	***N*** **(%)**	**OR (with 95% CI)**	***P***	**OR (with 95% CI)**	***P***	**OR (with 95% CI)**	***P***
No malignancy	683331 (93.3)	175952 (91.6)	1 (−)		1 (−)		1 (−)	
Malignancy (C00–43+45–96)	48704 (6.7)	16129 (8.4)	1.30 (1.27–1.33)	<0.001	1.29 (1.27–1.32)	<0.001	1.29 (1.27–1.32)	<0.001
Any solid (C00–43+45–80)	44979 (6.1)	13736 (7.2)	1.20 (1.18–1.23)	<0.001	1.19 (1.17–1.22)	<0.001	1.19 (1.16–1.22)	<0.001
Oral (C00–06)	298 (0)	102 (0.1)	1.41 (1.11–1.78)	0.007	1.41 (1.11–1.79)	0.006	1.40 (1.10–1.78)	0.007
Salivary (C07–08)	271 (0)	79 (0)	1.18 (0.91–1.55)	0.22	1.17 (0.89–1.53)	0.26	1.17 (0.89–1.53)	0.26
Oesophageal (C15)	359 (0)	131 (0.1)	1.42 (1.14–1.77)	0.002	1.41 (1.13–1.76)	0.003	1.39 (1.11–1.73)	0.005
Stomach (C16)	311 (0)	103 (0.1)	1.28 (1.00–1.62)	0.051	1.30 (1.02–1.66)	0.035	1.30 (1.02–1.65)	0.042
Colorectal (C18–20)	4805 (0.7)	1375 (0.7)	1.10 (1.03–1.18)	0.003	1.10 (1.03–1.17)	0.005	1.10 (1.03–1.18)	0.005
Larynx (C32)	318 (0)	75 (0)	0.89 (0.68–1.17)	0.41	0.87 (0.66–1.14)	0.3	0.86 (0.65–1.13)	0.28
Lung (C34)	1047 (0.1)	437 (0.2)	1.62 (1.44–1.83)	<0.001	1.50 (1.33–1.69)	<0.001	1.44 (1.27–1.62)	<0.001
Melanoma (C43)	2965 (0.4)	818 (0.4)	1.07 (0.98–1.16)	0.14	1.07 (0.98–1.16)	0.12	1.07 (0.99–1.17)	0.1
Breast (C50)	13520 (1.8)	4205 (2.2)	1.24 (1.20–1.29)	<0.001	1.24 (1.20–1.29)	<0.001	1.24 (1.20–1.29)	<0.001
Cervical (C53)	900 (0.1)	243 (0.1)	1.10 (0.95–1.29)	0.2	1.10 (0.95–1.28)	0.22	1.09 (0.94–1.27)	0.25
Uterus (C54–55)	1299 (0.2)	312 (0.2)	0.95 (0.83–1.08)	0.42	0.95 (0.83–1.09)	0.47	0.96 (0.84–1.09)	0.51
Ovarian (C56)	974 (0.1)	298 (0.2)	1.16 (1.01–1.34)	0.038	1.17 (1.01–1.34)	0.037	1.17 (1.01–1.35)	0.034
Prostate (C61)	5812 (0.8)	1690 (0.9)	1.15 (1.08–1.22)	<0.001	1.14 (1.08–1.21)	<0.001	1.13 (1.07–1.20)	<0.001
Testicular (C62)	385 (0.1)	127 (0.1)	1.16 (0.93–1.45)	0.19	1.16 (0.93–1.44)	0.2	1.15 (0.92–1.43)	0.22
Kidney (C64)	407 (0.1)	139 (0.1)	1.34 (1.09–1.64)	0.006	1.28 (1.05–1.57)	0.019	1.26 (1.03–1.55)	0.028
Bladder (C67)	2151 (0.3)	679 (0.4)	1.26 (1.15–1.38)	<0.001	1.24 (1.13–1.36)	<0.001	1.25 (1.14–1.37)	<0.001
CNS (C71–72)	248 (0)	159 (0.1)	2.28 (1.83–2.84)	<0.001	2.31 (1.85–2.88)	<0.001	2.12 (1.70–2.65)	<0.001
Thyroid (C73)	362 (0)	105 (0.1)	1.15 (0.92–1.45)	0.22	1.14 (0.91–1.43)	0.26	1.12 (0.89–1.41)	0.33
Other/unclear solid	8547 (1.2)	2659 (1.4)	1.22 (1.17–1.28)	<0.001	1.21 (1.15–1.27)	<0.001	1.20 (1.14–1.26)	<0.001
Any haematological (C81–96)	3725 (0.5)	2393 (1.2)	2.49 (2.35–2.64)	<0.001	2.46 (2.33–2.60)	<0.001	2.42 (2.28–2.56)	<0.001
Lymphoma (C81–86)	1979 (0.3)	1213 (0.6)	2.35 (2.17–2.54)	<0.001	2.32 (2.14–2.51)	<0.001	2.28 (2.11–2.47)	<0.001
Myeloma (C90)	356 (0)	367 (0.2)	4.27 (3.63–5.02)	<0.001	4.24 (3.60–4.99)	<0.001	4.05 (3.43–4.77)	<0.001
Leukaemia (C91–95)	1198 (0.2)	713 (0.4)	2.34 (2.11–2.59)	<0.001	2.31 (2.09–2.56)	<0.001	2.29 (2.07–2.54)	<0.001
Other haematological (C96/88)	192 (0)	100 (0.1)	1.83 (1.40–2.38)	<0.001	1.78 (1.36–2.32)	<0.001	1.75 (1.34–2.29)	<0.001

a adjusted for matching factors only, that is, age, sex, practice and calendar time.

b adjusted additionally for diabetes, SLE, IBD, RA, COPD, depression, asthma, renal failure, HIV, OID, inhaled corticosteroid treatment, BMI, smoking and alcohol use.

c Adjusted additionally for covariates potentially on the causal pathway that is, GP prescribed oral corticosteroids and other immunosuppression, and HSCT. See Supplementary Appendix B for estimates of the other covariates.
