# Supplementary material for: Herpes zoster risk after 21 specific cancers: population-based case–control study
Source: Br J Cancer. 2017 May 2;116(12):1643–51. doi: 10.1038/bjc.2017.124 (PMC5518853; doi:10.1038/bjc.2017.124)
Supplement: Supplementary Appendix B [file bjc2017124x2.doc]

**Protocol approved by ISAC (reference 16_113A)**

1. **Lay Summary (Max. 200 words)**

Cancer or a history of cancer could plausibly be linked to risk of zoster (known commonly as shingles) because both diseases can be affected by the health of the immune system, and this may be further affected by cancer treatments. Recent US data suggest a substantial increase in zoster risk among people with a previous cancer diagnosis. A detailed investigation of this in CPRD would help establish how overall/site-specific cancers are related to zoster risk. We will identify people with a zoster diagnosis, and others with similar general characteristics but no zoster diagnosis, and we will look back in the GP medical records of both groups to identify previous cancers, and to examine whether previous cancers were more likely among those with a zoster diagnosis. We will also account for differences in other important characteristics between the groups. The analysis will help us to understand whether cancer or its treatment is linked to an increased future risk of zoster; the results will be of interest to patients with cancer or a history of cancer and their doctors. Since a zoster vaccine is now available, the results may also help to inform future vaccination policies for zoster.

1. **Technical Summary (Max. 200 words)**

The objective of the study is to investigate the association between having had a cancer diagnosis and future risk of herpes zoster (also known as shingles). The two diseases could plausibly be associated because both have associations with immune function, which may be further affected by cancer treatments. Recent US data suggested a substantial increase in zoster risk among people with a previous cancer diagnosis. Our aim is to investigate this further by looking at how “any cancer” and common site-specific cancers (breast, colorectal, lung, prostate) are associated with future zoster risk, adjusting for potential confounders. We will conduct a matched case-control study: all individuals with an incident zoster diagnosis will be identified and matched on age, sex and GP practice to people who are under follow-up in CPRD at the same time but have no history of zoster. Conditional logistic regression models will be used to examine associations between cancer and zoster, both unadjusted, and adjusted for a range of potential confounders. The temporal relationship between cancer and zoster will also be examined by categorising the time between cancer diagnosis and index date. The results will be of interest to people with a history of cancer, and may help to inform zoster vaccination policy.

1. **Objectives, Specific Aims and Rationale**

*(i) The broad research objectives*

To investigate the association between overall and specific cancers, and risk of developing herpes zoster among adults.

*(ii) The specific aims; any hypotheses to be tested should be stated here.*

1. Determine if there is an increased risk of herpes zoster among adults with any previous cancer. *The null hypothesis is that there is no increased risk of herpes zoster among adults with a history of cancer as compared to the general population.*

2. Determine if the risk of zoster is increased with common specific malignancies (lung, breast, colon and prostate cancer). These cancer types are chosen based on having power available to detect clinically relevant effects (see section G). *The null hypotheses will be that that there is no increased risk of herpes zoster among adults with a history of these specific cancers as compared to the general population.*

3. Analyse the temporal relationship between the malignancy diagnosis and the risk of herpes zoster, in particular differences in risk between the immediate period post-diagnosis (when cancer treatment is likely to be ongoing) and later survivorship.

4. Investigate whether any associations between zoster risk and cancer are modified by age and sex.

5. Compare rates of postherpetic neuralgia (a common and painful complication of zoster) between people with and without a history of cancer, among those with a zoster diagnosis.

*(iii) An explanation of how achievement of the specific aims will further the research objectives*

The above aims are important components of the overall research objective, and meeting these aims will give us a more detailed understanding of the association between cancer and zoster risk. This information will be useful to people living with a history of cancer, and their clinicians, and could be used to guide future vaccination recommendations.

1. **Background**

Herpes zoster (commonly known as shingles) is caused by the reactivation of latent varicella zoster virus when specific cell mediated immunity becomes compromised. It is a common disease in older people,[^1^](#_ENREF_1) with a lifetime risk of up to 30% rising to 50% among those living to 85 years.[^2^](#_ENREF_2) Zoster typically presents as a painful unilateral vesicular dermatomal rash that causes acute morbidity lasting two to four weeks.[^3^](#_ENREF_3) A severe complication—postherpetic neuralgia—although uncommon in patients aged under 50, develops in 12% of zoster patients aged 50 years or over; it causes intense pain that can last from months to years and is associated with considerable impairment of quality of life.

People with a past diagnosis of cancer might plausibly have an increased risk of zoster, because both diseases can be associated with depleted immune function; certain cancer treatments may also have short- and/or long-term impacts on immune function. A recent US study looked at zoster risk in around a million cancer patients aged over 65 years, identified in the US Surveillance, Epidemiology, and End Results (SEER) cancer registry, and a random group of non-cancer Medicare patients.[^5^](#_ENREF_5) Both solid and haematological cancers were associated with an increased risk of zoster in this elderly patient population.

In previous work (protocol 11_028A2R), we have investigated a broad range of potential risk factors for zoster.[^6^](#_ENREF_6) In that study we adjusted for recent haematological malignancies, but we did not investigate cancer as a risk factor of interest. We now wish to build on both our earlier study, and the US findings mentioned above, by investigating the associations between cancer and zoster in a UK primary care population, by further exploring the temporal relationship between cancer and zoster risk, and by comparing rates of postherpetic neuralgia (a common and painful complication of zoster) between zoster patients with and without a history of cancer.

1. **Study Type**

Hypothesis testing.

1. **Study Design**

Case-control study. This is a well-established and valid study design for assessing associations between proposed risk factors and outcomes, and lends itself naturally to investigation of multiple exposures (namely any cancer, and site-specific cancers). We also choose this design over other options partly for pragmatic reasons, as it allows us to re-use programming code from a previous case-control study which looked at a wide range of risk factors for zoster,[^6^](#_ENREF_6) which we can adapt to extract our new study population and covariates. This ensures maximum value from the academic resources going into the project.

1. **Sample Size**

We conducted a feasibility count using a similar previous case-control dataset which used data to end 2011.[^6^](#_ENREF_6) This dataset included 144,959 zoster cases, with 11,423 in the year 2011. 59% of cases were women. Using data for another 20 months to August 2013 (see section I) would therefore be expected to add (20/12*11423 =) ~ 20,000 cases, making 165,000 zoster cases in total.

The proportion of controls with any cancer prior to the index date in the previous dataset was 6.3%, and the percentages for specific common cancers were 3.5% for breast cancer (among women), 0.8% for colorectal cancer, 0.1% for lung cancer, and 1.7% for prostate cancer (among men).

Using the “power mcc” routine in Stata for calculating power/sample size in matched case-control studies, these inputs lead to the following minimum detectable odds ratios, with alpha = 0.05 and power of 80%: 1.03 for any cancer, 1.06 for breast cancer, 1.09 for colorectal cancer, 1.26 for lung cancer, and 1.09 for prostate cancer. We therefore conclude that we will have sufficient power to detect clinically relevant effects for these outcomes.

1. **Data Linkage Required (if applicable)**

None required. We are not requesting linkage to cancer registry for cancer ascertainment because CPRD has a high positive predictive value and sensitivity for detecting nationally registered cancers, so we do not feel the financial and time costs associated with linkage would be justified. We are not requesting linkage to Hospital Episodes Statistics to improve zoster ascertainment because in a previous study in which we did take this approach, 99.5% of zoster cases were identifiable using primary care data alone.[^6^](#_ENREF_6)

1. **Study Population**

The base study population will consist of all patients aged 18 years or over, under follow-up between 1 January 2000 and 31 August 2013, with no evidence of previous zoster (no codes for zoster or postherpetic neuralgia before the start of follow-up). The end-date of August 2013 is chosen to avoid the period of time after zoster vaccination was rolled out in the UK, since there might be differential uptake of zoster vaccine between people with and without a history of cancer, introducing potentially important confounding. Within this base population, we will identify zoster *cases* as those with a zoster Read code (see code list in Appendix 1) and at least 12 months’ follow-up before a first diagnosis of zoster, to exclude past cases of zoster recorded retrospectively after registration at a general practice.

1. **Selection of comparison group(s) or controls**

We will identify up to four controls per case at random using incidence density sampling, matched on practice, sex, and age (within 1 year), and with no history of zoster or postherpetic neuralgia at the index date of the case. Controls will be required to be registered with the practice at the index date of the case and for at least 12 months before, and to be active in the database (defined as at least one consultation record of any kind, including repeat prescriptions and face to face or telephone consultations, in the period from 6 months before to 12 months after the index date). Controls will take the index date of their matched case in the final dataset.

1. **Exposures, Outcomes and Covariates**

Cancers (all cancer, and the following site-specific cancers: breast, colorectal, lung, prostate) will be identified through searching the patient primary care records for Read codes indicating cancer. A comprehensive list of these Read codes has already been developed for a recent major project and the methods are detailed in that publication[^9^](#_ENREF_9) with the key parts reproduced in Appendix 2 to this protocol. The previous code list will be updated to be current with the latest Read code dictionary version.

Potential confounders will be identified through the patient primary care records and will consist of smoking, alcohol consumption, BMI, HIV, other unspecified cellular immunodeficiencies, rheumatoid arthritis, inflammatory bowel disease, chronic kidney disease, chronic obstructive pulmonary disease, asthma, systemic lupus erythematosus, depression and diabetes (by type). The role of corticosteroid use and haematopoietic stem cell transplantation will be examined separately since these may be on the causal pathway between cancer and zoster risk. The aforementioned variables are defined as in our previous study of zoster risk factors.[^6^](#_ENREF_6)

For the last objective, postherpetic neuralgia will be defined as in previous work,[^10^](#_ENREF_10) as outlined in Appendix 3.

1. **Data/ Statistical analysis**

We will describe the characteristics of the study population by case-control status. Conditional logistic regression will be used for the analysis, which accounts for the matched design. All odds ratios will therefore account for the matching factors of age (within 1 year), sex, practice, and calendar time. Initially, we will calculate odds ratios for the association between cancer and odds of zoster without further adjustment. We will then adjust for all covariates listed in Section K.

To examine the temporal relationship between cancer and zoster, we will divide exposure into a multi-category variable distinguishing between recent and historical cancer diagnoses (split initially into 0-6 months, 7-12 months, 1-4 years, >=5 years before index; categories may be collapsed if appropriate – i.e. if ORs are similar in adjacent categories). All models will be fitted separately for the 5 cancer exposures to be considered, namely all cancer, breast cancer, colorectal cancer, lung cancer, prostate cancer. For breast cancer we will restrict the study population to women, and for prostate cancer to men.

Modification of associations by age and sex (for non sex-specific cancers) will be assessed by fitting interaction terms in our models and conducting likelihood ratio tests on nested models with and without the interaction terms.

Associations between cancer and postherpetic neuralgia will be examined within zoster patients only. For this aim, the zoster group will be treated as a cohort, and we will calculate crude and adjusted odds ratios for the association between cancer/specific cancers and zoster risk, using logistic regression.

1. **Plan for addressing confounding**

As in Sections J, K and L we will attempt to reduce confounding by a wide range of factors by a combination of matching, and adjustment for multiple covariates as listed.

1. **Plan for addressing missing data**

We plan to use complete case analysis to allow for missing data in the smoking, BMI and alcohol data. This assumes that the probability of being a complete case is independent of the outcome (zoster), conditional on covariates.[^11^](#_ENREF_11) We do not think that the assumptions required for multiple imputation would hold for these variables, since missingness of BMI/smoking/alcohol may well depend on the underlying values of those variables (e.g. obese people are more likely to have BMI recorded).

1. **Limitations of the study design, data sources and analytical methods**

A possible limitation is misclassification of zoster status. There has not been any specific validation of zoster diagnoses in CPRD. The key differential diagnosis for zoster is recurrent herpes simplex, which occasionally presents with a dermatomal distribution. Viral culture and PCR are highly unlikely to have been performed in routine practice and would be cost prohibitive to measure in a large population-based study. However, zoster is usually a straightforward clinical diagnosis based on classical clinical presentation.

Additional limitations are that documentation in GP records of clinical presentation or severity of zoster is likely to be limited, as is information on cancer stage and timing and types of treatment.

1. **Patient or user group involvement (if applicable)**

None planned

1. **Plans for disseminating and communicating study results, including the presence or absence of any restrictions on the extent and timing of publication**

We plan to present the findings at scientific conferences, and to publish in peer review journals.

**AMENDMENT – 7^th^ September 2016 (approved by ISAC)**

Broadening of analysis to consider more specific cancer sites

We will consider associations with a broader range of the most common site-specific cancer diagnoses than originally mentioned in the protocol, namely oral (ICD-10 code C00-06), salivary(C07-08), oesophageal (C15), stomach (C16), colorectal (C18-20), larynx (C32), lung (C34), melanoma (C43), breast (C50), cervical (C53), uterus (C54-55), ovarian (C56), prostate (C61), testicular (C62), kidney (C64), bladder (C67), brain/CNS (C71-72), thyroid (C73), lymphoma (C81-85), myeloma (C90), leukaemia (C91-95). Read codes have been mapped to ICD headings as part of previous work (reference 9) and this mapping will be used to define the broader range of site-specific cancers. In addition, we will look at associations with the following groupings of cancers: any malignancy, any solid malignancy (C00-C75), any haematological malignancy (C81-96).

The reason for this change is that during ongoing discussions about the project, it was decided that looking at a broader range of specific malignancies than originally specified would help to elucidate patterns and differences in the associations between different cancer histories and zoster risk.

*Power considerations*

There are 192,081 zoster cases in the dataset (note, this is higher than we estimated in the original protocol). Considering the site-specific cancers above, the least common are oral, salivary and stomach cancers (each with prevalence 0.04% among controls). Using the same sample size methodology as used in the protocol, this gives a detectable odds ratio of 1.38, with power=0.8 and alpha=5% for each of these specific malignancies. For all other site-specific cancers we will be able to detect smaller odds ratios. This suggests that, despite lower power for some cancers, the numbers are sufficient to contribute useful information on the associations with all cancers, both immediately and as part of possible future meta-analyses. We will treat estimates with wide confidence intervals with appropriate caution.

1. **References**

1. Hope-Simpson RE. Postherpetic neuralgia. *J R Coll Gen Pract* 1975; **25**(157): 571-5.

2. Thomas SL, Hall AJ. What does epidemiology tell us about risk factors for herpes zoster? *Lancet Infect Dis* 2004; **4**(1): 26-33.

3. Gnann JW, Jr., Whitley RJ. Clinical practice. Herpes zoster. *N Engl J Med* 2002; **347**(5): 340-6.

4. Oxman MN, Levin MJ, Johnson GR, et al. A vaccine to prevent herpes zoster and postherpetic neuralgia in older adults. *N Engl J Med* 2005; **352**(22): 2271-84.

5. Yenikomshian MA, Guignard AP, Haguinet F, et al. The epidemiology of herpes zoster and its complications in Medicare cancer patients. *BMC Infect Dis* 2015; **15**: 106.

6. Forbes HJ, Bhaskaran K, Thomas SL, Smeeth L, Clayton T, Langan SM. Quantification of risk factors for herpes zoster: population based case-control study. *BMJ* 2014; **348**: g2911.

7. Bhaskaran K, Williams R, Ranopa M. Scope, completeness and concordance of national cancer registration data linked to CPRD primary care data. Farr Institute International Conference. St Andrews, Scotland: Abstract C2_1405; 2015.

8. Boggon R, van Staa TP, Chapman M, Gallagher AM, Hammad TA, Richards MA. Cancer recording and mortality in the General Practice Research Database and linked cancer registries. *Pharmacoepidemiol Drug Saf* 2012; **22**(2): 168-75.

9. Bhaskaran K, Douglas I, Forbes H, dos-Santos-Silva I, Leon DA, Smeeth L. Body-mass index and risk of 22 specific cancers: a population-based cohort study of 5.24 million UK adults. *Lancet* 2014; **384**(9945): 755-65.

10. Forbes H, Bhaskaran K, Thomas SL, et al. Understanding risk factors for herpes zoster and postherpatic neuralgia in UK primary care: investigations to inform vaccine policy. *Neurology* 2016; **(in press)**.

11. White IR, Carlin JB. Bias and efficiency of multiple imputation compared with complete-case analysis for missing covariate values. *Stat Med* 2010; **29**(28): 2920-31.

**Protocol appendix 1 – code list for zoster**

| **medcode** | **readterm** |
| --- | --- |
| 390 | Herpes zoster |
| 516 | Shingles |
| 7331 | Ramsey Hunt Syndrome |
| 8936 | Ophthalmic herpes zoster infection |
| 14718 | Herpes zoster with ophthalmic complication |
| 14793 | Herpes zoster otitis externa |
| 18918 | Herpes zoster ophthalmicus |
| 21069 | Herpes zoster with unspecified complication |
| 21471 | Herpes zoster NOS |
| 25320 | Herpes zoster with dermatitis of eyelid |
| 27403 | Geniculate herpes zoster |
| 27546 | Herpes zoster with keratoconjunctivitis |
| 31681 | Herpes zoster - otitis externa |
| 33810 | Herpes zoster with other ophthalmic complication |
| 38531 | Herpes zoster with other specified complication NOS |
| 39692 | Polyneuropathy in herpes zoster |
| 43235 | Herpes zoster with other specified complication |
| 44944 | Herpes zoster with meningitis |
| 47375 | Zoster encephalitis |
| 50537 | Herpes zoster with other CNS complications |
| 51692 | Encephalitis due to herpes zoster |
| 52126 | Herpes zoster with other central nervous system complication |
| 52319 | Disseminated zoster |
| 55940 | Herpes zoster iridocyclitis |
| 57895 | Herpes zoster meningitis |
| 62558 | Infective otitis externa due to herpes zoster |
| 63739 | Herpes zoster with other CNS complication NOS |
| 69405 | Herpes zoster encephalitis |
| 70197 | [X]Zoster without complications |
| 71464 | Meningitis due to herpes zoster virus |

**Protocol appendix 2 - Identification of cancer cases in CPRD**

**(extract from appendix to Bhaskaran et al, Lancet 2014 – an updated version of the resulting code list - which has already been mapped to ICD-10 chapter 2 headings to determine cancer site - will be used for the present study)**

To identify cancers in CPRD, the dictionary of Read codes (used by GPs to record clinical diagnoses) was systematically searched to find cancer-related codes using the keywords/word fragments below. The codes picked up by this search were then screened and those indicating malignancy were identified and classified by cancer type (done by KB, reviewed by LS). Each patient’s record was then searched for these cancer codes. The earliest code for a particular cancer type was taken as the date of diagnosis.

Words and word fragments used to search Read code dictionary for cancer-related terms:

MELANOMA NEOP TUMOUR CANCER MALIG CARCINOM LEUKAEM METASTA SARCOM LYMPHOM HODGKIN ACROSPIROMA ADAMANTINOMA ADENOACANTHOMA ADENOCARCIONOMA ADENOMA ADENOMATOSIS ANGIOENDOTHELIOMA ANGIOENDOTHELIOMATOSIS ANGIOMYXOMA APUDOMA ARGENTAFFINOMA ARYTHREMIA ASTROCYTOMA BLASTOM BOWEN BURKITT CARCINOID CHEMODECTOMA CHEMOTHERAPY CHLOROMA CHOLANGIOMA CHONDROMATOSIS CHORDOMA CRANIOPHARYNGIOMA CYSTADENOMA DESMOID ECCHONDROSIS EPENDYMOMA EPITHELIOMA ERYTHRAEMIA ERYTHRAEMIA ERYTHRAEMIA ERYTHREMIA ERYTHROPLASIA FIBROMA GAMMOPATHY GASTRINOMA GERMINOMA GLEASON GLIOMA GLUCAGONOMA HAEMANGIOENDOTHELIOMA HAEMANGIOENDOTHELIOMA HEMANGIOENDOTHELIOMA HEPATOMA HISTIOCYTIC HISTIOCYTOMA HISTIOCYTOSIS HYDATIDIFORM HYPERNEPHR HYPERNEPHR IMMUNOPROLIFERATIVE IMMUNOPROLIFERATIVE INSULINOMA KAHLER LEIOMYOMATOSIS LETTERER LYMPHANGIOMYOMATOSIS LYMPHOM LYMPHOPROLIFERATIVE MASTOCYTOMA MASTOCYTOSIS MECKEL MENINGIOMA MESENCHYMOMA MESONEPHROMA MESOTHELIOMA MESOTHELIOMA MYELODYSPLASTIC MYELOFIBROSIS MYELOMA MYELOMA MYELOPROLIFERATIVE MYELOSCLEROSIS MYELOSIS NEPHROMA NEURILEMMOMA NEURINOMATOSIS NEUROCYTOMA NEUROFIBROMATOSIS OSTEOCLASTOMA PAGET PANCOAST PANMYELOSIS PARAGANGLIOMA PERICYTOMA PINEALOMA PINEOCYTOMA PLASMACYTOMA PLASTICA POLYCYTHAEMIA POLYCYTHEMIA POLYEMBRYOMA PSEUDOMYXOMA RADIOTHERAPY SEMINOMA SEZARY TERATOMA TERATOMA THECOMA THROMBOCYTHAEMIA THROMBOCYTHEMIA THYMOMA VIPOMA WALDENSTROM [M] “ANGIOIMMUNOBLASTIC LYMPHADENOPATHY” “ATYPICAL FIBROXANTHOMA” BRILL CA CA-IN-SITU “DI GUGLIELMO” “GIANT PIGMENTED NAEVUS” “GIANT PIGMENTED NEVUS” “HEAVY CHAIN” “HUTCHINSON'S MELANOTIC” “MAST CELL” “MYCOSIS FUNGOIDES” "NEO/" “REFRACTORY ANAEMIA” “REFRACTORY ANEMIA” “RODENT ULCER” “STROMAL MYOSIS” “STRUMA OVARII” “TRANSITIONAL CELL PAPILLOMA, INVERTED” “UROTHELIAL PAPILLOMA”

**Protocol appendix 3 – Definition of postherpetic neuralgia (PHN)**

PHN will be defined in the main analysis as diagnosed, probable or possible PHN, as below. This is the same definition as used in our previous work for Forbes et al, Neurology 2016 (in press).

***Diagnosed PHN***

PHN code 90‐365 days post‐zoster

| CPRD medical code | description |
| --- | --- |
| 1598 | Post-herpetic neuralgia |
| 7584 | Post-herpetic trigeminal neuralgia |
| 10223 | Postherpetic neuralgia |
| 11498 | ZERIDAME SR tablets 150mg [ACTAVIS] |
| 17180 | Postzoster neuralgia |
| 31709 | Postherpetic polyneuropathy |

***Probable PHN***

Zoster code and prescription consistent with PHN• on same day (90‐365 days post‐zoster)

Non‐specific neuralgia code (90‐365 days post‐zoster)

**NEW** anticonvulsant or capsaicin cream or lidocaine patch prescription (90‐180 days postzoster)

**NEW** tricyclic antidepressants 90‐180 days post‐zoster with no other indication on the day of

the prescription, plus evidence of the drug being prescribed for zoster or PHN previously†

***Possible PHN***

**NEW** tricyclic antidepressants 90‐180 days post‐zoster with no other indication on the day of

the prescription

**NEW** strong painkiller 90‐180 days following zoster with no other indication on the day of the

prescription, plus evidence of the drug being prescribed for zoster or PHN previously†

Non‐specific neuropathic pain code (90‐365 days post‐zoster)

Notes:

•Prescriptions included anticonvulsants, tricyclic antidepressants, capsaicin cream or lidocaine patch.

**NEW** prescriptions were defined as no previous prescriptions of the same medication type 12 months to two weeks prior to

zoster, to increase the likelihood of the medication being prescribed for PHN (medications may have been prescribed for pain

management two weeks pre‐zoster, if zoster initially presented without a rash).

†Here, previously is defined as a prescription 0‐89 days following zoster.

Medications indicative of zoster were only considered in the 90‐180 day period after zoster diagnosis (rather than 90‐365 days) to reduce the chance of misclassifying other reasons for medication use as PHN.

**Summary of deviations from the protocol and justification**

1) In the approved protocol, we planned to restrict all analyses to data before August 2013, to avoid the period of time after which zoster vaccination was rolled out in the UK. We later decided to maximize available power by including all data to end of CPRD data collection. To check that the use of data after the rollout of zoster vaccination did not impact on our results, we added a sensitivity analysis which right censored follow-up at pre-August 2013 as had been originally planned.

2) We added a sensitivity analysis with further adjustment for GP-prescribed aciclovir use, in response to a peer reviewer comment.

3) We added a post-hoc analysis separating leukaemia into acute/chronic/other types, in response to a peer reviewer comment.

The above changes were considered “minor” changes from the protocol not requiring formal approval, following ISAC guidance on the issue of protocol deviations and amendments.
