# Supplementary material for: Herpes zoster risk after 21 specific cancers: population-based case–control study
Source: Br J Cancer. 2017 May 2;116(12):1643–51. doi: 10.1038/bjc.2017.124 (PMC5518853; doi:10.1038/bjc.2017.124)
Supplement: Supplementary Appendix I [file bjc2017124x9.pdf]

Appendix I: Estimates for adjustment variables in the adjusted "all cancer" model (see Table 2, model 2 and 3)

	Adjusted[1] OR (95% CI)	Adjusted[2] OR (95% CI)
**Comorbidities and lifestyle factors**		
Systemic Lupus Erythematosus	1.65 (1.48-1.85)	1.46 (1.3-1.63)
Inflammatory Bowel Disease	1.32 (1.25-1.38)	1.22 (1.16-1.28)
Rheumatoid Arthritis	1.51 (1.45-1.58)	1.21 (1.16-1.27)
Chronic obstructive pulmonary disease	1.25 (1.21-1.29)	1.21 (1.17-1.24)
Depression	1.15 (1.12-1.18)	1.15 (1.12-1.18)
Asthma	1.11 (1.07-1.14)	1.11 (1.08-1.15)
Chronic kidney disease	1.13 (1.1-1.16)	1.11 (1.09-1.14)
HIV	4.05 (3.17-5.17)	4.02 (3.15-5.14)
Other unspecified cellular immune deficiencies	1.46 (1.17-1.83)	1.35 (1.08-1.69)
Inhaled corticosteroid treatment	1.12 (1.09-1.15)	1.07 (1.04-1.1)
Diabetes		
Type 1	1.13 (1-1.27)	1.12 (0.99-1.25)
Type 2	1 (0.98-1.02)	1 (0.98-1.02)
Unknown	1.25 (1.16-1.35)	1.24 (1.15-1.34)
BMI category		
Underweight	1 (-)	1 (-)
Normal Weight	1.03 (0.99-1.07)	1.03 (0.99-1.08)
Overweight	1.04 (1-1.08)	1.04 (1-1.09)
Obese	1.04 (0.99-1.08)	1.04 (1-1.08)
Smoking		
Never-smoker	1 (-)	1 (-)
Current smoker	1.06 (1.04-1.08)	1.06 (1.04-1.08)
Ex-smoker	1.09 (1.07-1.12)	1.09 (1.07-1.12)
Alcohol use		
Never-drinker	1 (-)	1 (-)
Current drinker	0.91 (0.9-0.93)	0.91 (0.9-0.93)
Ex-drinker	1.06 (1.04-1.07)	1.06 (1.04-1.07)
**Potential cancer treatments**		
Other immunosuppressive treatment		1.61 (1.53-1.71)
Haematopoietic stem cell transplantation		2.68 (1.63-4.42)
Oral corticosteroid treatment		1.51 (1.46-1.56)

Note. Patients may have many comorbidities and/or treatments, comparisons are to those not having the specified condition or treatment. 1. Adjusting for all covariates in table except HSCT, oral corticosteroid and other immunosuppressant treatment and the most detailed malignancy classification. 2. Adjusting additionally for HSCT, oral corticosteroid and other immunosuppressant treatment.
